# Experimental Study of Burn Damage Progression in a Human Composite Tissue Model

**DOI:** 10.3390/biology10010040

**Published:** 2021-01-08

**Authors:** Dandan Hao, Miao Qu, Mahtab Nourbakhsh

**Affiliations:** Department of Geriatric Medicine, RWTH Aachen University Hospital, 52074 Aachen, Germany; dhao@ukaachen.de (D.H.); qumiao2014@gmail.com (M.Q.)

**Keywords:** human model, thermal burns, damage progression, inflammation: tissue-resident macrophages, fibroblasts

## Abstract

**Simple Summary:**

Patients with severe burn injuries undergo surgical procedures to remove dead skin and underlying tissue. The study of tissue damage inflicted by burns is vital for further improving current treatments. Numerous animal burn models have been employed to inflict burns at different temperatures and study the local reaction of harmed tissue and its consequences for the whole organism. Because human skin tissue is different than that of animals, we used human skin tissue discarded from surgeries and exposed the tissue to different burn injuries. The samples were maintained for seven days to study the course of burn damage and activation of different cells in human skin tissue. Our study shows that milder burns result in slowly expanding damage, while severe burns cause deep persistent damage to the skin and underlying fat tissue. These different patterns were also reflected by the activation of immune and repair cells within the fat tissue. Our study suggests that fat tissue adjacent to burn wounds can play a crucial role in tissue repair. Thus, examination of fat tissue vitality before surgery may improve outcomes.

**Abstract:**

Comparative studies of human tissue damage caused by burns are challenging because precise information regarding the temperature, time, and duration of the exposure is often missing. Animal models cannot be fully translated to the human system due to interspecies differences in cutaneous tissues. We used a human composite tissue model to compare tissue damage caused by thermal burns with different dynamics. Equal subcutaneous/cutaneous composite tissue samples from six donors were first exposed to either preheated steel (100 °C) or a precision flame burner (300 °C) and were then maintained in vitro for seven days. Histological and immunohistochemical analyses revealed that flame burns instantly caused deep and stable damage to the subcutaneous tissue, which stayed constant for seven days. By contrast, contact burns inflicted tissue damage that was initially superficial but then expanded deeper into the adipose tissue. This spatiotemporal expansion of tissue damage was essentially accompanied by macrophage and fibroblast activation, which points towards inflammation resolution and wound healing. Our study suggests that thermal differences in burns directly influence the course of tissue damage, the cellular response and, consequently, the likely dynamics of repair processes days after burn injuries.

## 1. Introduction

The severity of burn injuries is related to the rate at which heat is transferred from the heating agent to the skin. Thermal burns account for the majority of human burn injuries and include contact and flame burns bearing different temperature transmission profiles. Contact burns often result from direct contact with a hot object and are confined to the part of skin that touched the hot object, whereas flames usually affect larger areas by indirect transmission of heat [1]. Our current knowledge of local tissue damage by burns has been obtained using animal burn models. However, heat transmission properties of human cutaneous tissue have not yet been studied experimentally.

Thermal burns mostly involve the cutaneous and subcutaneous adipose tissues that direct a local inflammatory response, which not only protects against pathogens but also promotes tissue formation and remodeling [2,3]. In particular, subcutaneous adipose tissue contains numerous macrophages, fibroblasts and stem cells that play significant roles in the initiation, elongation and termination of inflammation [4].

Tissue-resident macrophages emerge during embryonic development and persist in many tissues via self-renewal. They are capable of this by local proliferation and produce a variety of factors that stimulate the activation, proliferation, and differentiation of many cells, including fibroblasts and stem cells [5,6]. Earlier studies with cultured human bone marrow-derived macrophages indicated that two main phenotypes of macrophages can be induced in vitro: type M1, which expresses proinflammatory cytokines and type M2, which expresses anti-inflammatory cytokines [7]. This classification has provided a useful system to distinguish macrophages using a few non-overlapping markers. Animal studies revealed that macrophage classification may be more complex during acute injury, when tissue-resident and monocyte-derived macrophages coexist at the site of injury [8]. Thus, the classification of macrophages in M1 and M2 is particularly useful in well-defined in vitro conditions.

Following injury, subcutaneous resident macrophages function as sentinels and become rapidly activated and contribute to the local proinflammatory environment within the wound [9,10]. They secrete and respond to a number of different cytokines and chemokines to attract different sets of immune cells to the injury site, thereby affecting the proliferation and differentiation of stem cells. In addition, the interaction between resident macrophages and fibroblasts was hypothesized to mediate important signals towards wound healing [11]. As M1/M2 polarization governs tissue damage, the reversibility from both states is considered to have a critical therapeutic value but has not been established in reproducible human tissue models in vitro [12].

To study subcutaneous resident macrophage polarization in response to flame burns, we established a 3-dimensional human composite tissue model consisting of hypodermis and subcutaneous tissue [13]. A series of flame burn experiments previously confirmed the utility of our model for the study of local cellular responses to burn damage [13]. In the present study, we used this model to compare the spatiotemporal activation of subcutaneous macrophages and fibroblasts in response to controlled contact burns at 100 °C and flame burns at 300 °C bearing different temperature transmission profiles.

## 2. Materials and Methods

### 2.1. Tissue Specimens

Human composite subcutaneous/cutaneous tissue samples were obtained from 6 healthy donors undergoing elective surgery at the RWTH Aachen University Hospital as follows: donor 1, 31 years old, male, breast; donor 2, 44 years old, female, breast; donor 3, 41 years old, female, breast; donor 4, 27 years old, male, abdomen; donor 5, 49 years old, male, abdomen; and donor 6, 36 years old, female, abdomen. Ethical approval for the use of discarded surgical tissues was obtained from the Independent Ethics Committee of RWTH Aachen University Hospital and registered under identification number 206/13 on 11 December 2013. Donor consent was obtained before surgery according to the regulations of the Independent Ethics Committee of RWTH Aachen University Hospital.

### 2.2. In Vitro Burn Experiments

Tissue samples from every patient were cut into 2 equal pieces of cutaneous surface (2 × 2 cm) that included a 1-cm-thick layer of underlying subcutaneous fat tissue that did not reach the superficial fascia. The processed tissue pieces from every patient were subjected to contact or flame burns (Supplementary Figure S1). For contact burns, the composite tissue pieces were placed on a culture dish and burned on day 0 using a stainless steel cylindrical bar of 10-mm-diameter and 10-mm-length (1.9 g) for 30 s without extra pressure; the bar was preheated to 120 °C using an alcohol burner (RSG Solingen, Germany), and its temperature was continuously monitored using a thermocouple. Then, the steel bar was moved away from the gun flame and placed on the tissue as soon as it was cooled down to 100 °C. This was applied three times on the same position of the tissue surface, which was labeled using a surgical marking pen before burn injuries were inflicted. All contact burns were performed using the same stainless steel bar. For flame burns, the composite tissue pieces were burned on day 0 using a pre-adjusted stable flame surface at 300 °C for 15 s produced by a McKenna burner with adjustable air and fuel flow and integrated thermocouple on flame surface. The setup of the burner was designed to allow a homogeneous gas mixture past the cooled sintered metal matrix with a diameter of 60 mm, thereby providing a one-dimensional flow field at the burner exit and a well-defined flat flame downstream of the matrix. A cool water stream was introduced through an annular ring around the burner matrix to avoid any environmental air entrainment into the flame. Under these conditions, the McKenna burner was able to repeatedly produce highly stable and well-defined temperatures. Furthermore, a unique steel holder with a 10-mm-diameter orifice was fabricated to place the human tissue at an adjustable distance from the flame. During the experiments, the holder orifice was concentrically aligned with the burner. Therefore, all of the inflicted burn wounds in this study were of equal shape and size. The container also included a mount for a thermocouple at a distance of 1 mm from the tissue sample to detect the ambient gas temperature. Before each experiment, the holder position was adjusted to achieve 300 °C at a distance 1 mm from the epidermis. The cutaneous surface of the tissue samples was completely protected, except for a circle with a diameter of 1 cm that was exposed to the flame for a precise time frame. During contact and flame burn experiments, the temperature at the bottom of the tissue was measured between 20 to 25 °C. After inflicting burn injuries, human composite pieces bearing one burned zone (~0.785 cm^2^) and the distal posterior unburned zone (~3.215 cm^2^) surrounding the burn were obtained.

### 2.3. Human Composite Tissue and 3D Culture In Vitro

After burn injuries were inflicted, the tissue samples were embedded in a polysaccharide polymer (agarose) for extended maintenance in vitro. The embedding procedure consisted of 2 consecutive steps: sealing using superficial dehydration and embedding in a precise gradient of ultrapure polysaccharide polymer LowMelt-Agarose (Bio-Budget, Krefeld, Germany) in DMEM/F-12 cell culture medium (Gibco Life Technologies, Grand Island, NY, USA) supplemented with 10% fetal bovine serum (FBS, PAA, Cölbe, Germany) and penicillin and streptomycin (100 U/mL each; Sigma-Aldrich, Munich, Germany). The sealing step ensures direct contact with the embedding matrix. For embedding, a thin layer of agarose-medium mixture (1–1.5 mL) was added to the well of a 6-well culture plate. Following polymerization for 2 min, the tissue was mounted on the top of the first layer and surrounded by agarose-medium mixture to reach, but not cover the tissue surface. After complete polymerization of 5 min, 0.5 mL of fresh medium was added to form a thin layer and cover the tissue surface. The embedded sections were then incubated at 37 °C with 5% CO_2_, and the medium on the top of the polymer was replaced at days 3 and 6. At days 3, 5, and 7 after burning, the human tissue pieces were carefully separated from the embedding material and preserved in a freshly prepared mixture of methanol (VWR, Radnor, PA, USA), chloroform (Carl Roth, Karlsruhe, Germany), and acetic acid (Carl Roth, Karlsruhe, Germany) at a ratio of 6:3:1 for 24 h.

### 2.4. Hematoxylin and Eosin Staining

The preserved tissue pieces were then dehydrated, embedded in paraffin and sectioned into 3-μm sections using a SLIDE4003E microtome (pfm medical, Cologne, Germany) before staining. The sections were fixed on glass slides, deparaffinized and stained using a standard hematoxylin and eosin (HE) staining protocol (Merck Millipore, Darmstadt, Germany). Briefly, the procedure included nuclear staining with hematoxylin for 5–10 min, rinsing in warm water for 10 min, staining in 0.3% eosin for 5 min, rinsing with tap water, dehydrating in an ethanol gradient (80%, 96%, and 100%), treating with xylene, and sealing.

### 2.5. DAPI (4′,6-Diamidino-2-phenylindole) Staining

Embedded tissue sections were carefully separated from the embedding material and were fixed and sectioned as described for HE staining. The sections were deparaffinized, washed with PBS, treated with protein blocking solution (Dako, Hamburg, Germany) for 30 min at room temperature, and stained for DAPI (final concentration 1:100,000) for 10–15 min according to the manufacturer’s protocol (Dako, Hamburg, Germany). After they were rinsed 3 times with PBS, the sections were sealed with Mowiol (Sigma-Aldrich, Munich, Germany) and maintained at 4 °C overnight. Finally, sections were photographed under a fluorescence microscope (DM microscope 600).

### 2.6. Immunohistochemistry/Light Microscopy

Tissue sections subjected to HE staining or immunohistochemistry were analyzed under a light microscope. For immunohistochemistry, CD68, CD80, CD163, and HSP47 were chosen as the protein markers of subcutaneous monocytes, M1 macrophages, M2 macrophages and fibroblasts, respectively (Ito and Nagata 2017, Jaguin et al., 2013, Vogel et al., 2014). Embedded tissue pieces were sectioned into 3-μm sections, which were then deparaffinized according to the manufacturer’s protocol (Acris, Los Angeles, CA, USA) before they were incubated with a 1:250 dilution of the following primary antibodies: mouse anti-human CD68 (Agilent Technologies, Waldbronn, Germany), rabbit anti-human HSP47 (Abcam, Berlin, Germany), mouse anti-human CD80, and rabbit anti-human CD163 (Acris, Los Angeles, CA, USA). After the sections were treated with the 1:250 dilution of horseradish peroxidase-conjugated anti-mouse or anti-rabbit secondary antibodies (Medac, Wedel, Germany), the stain was developed with diaminobenzidine (DAB, Agilent Technologies, Waldbronn, Germany).

Macroscopic changes were viewed under a DM microscope 600 (Leica, Wetzlar, Germany), and images were processed using DISKUS software (Leica, Wetzlar, Germany). The damage depth was defined as the distance from the most superficial core of the burn site to the most distal visible tissue damage. Semi-quantitative analysis was performed with at least 5 randomly selected fields per slice at high magnification (400×) to calculate the number of positively stained cells within a photographic area (approximately 0.073 mm^2^), which was determined in at least 5 equivalent microscopic fields in different locations within the burned and unburned subcutaneous zones at the indicated days postburn.

### 2.7. Statistical Analysis

All results are reported as the mean ± standard deviation (SD). Statistical analysis was performed with SPSS 18.0. Results were compared using two-way repeated ANOVA with Bonferroni’s multiple comparisons. Differences with a *p* value ≤ 0.05 were considered statistically significant.

## 3. Results

### 3.1. Differential Progression of Tissue Damage by Contact and Flame Burns

**Figure 1:**
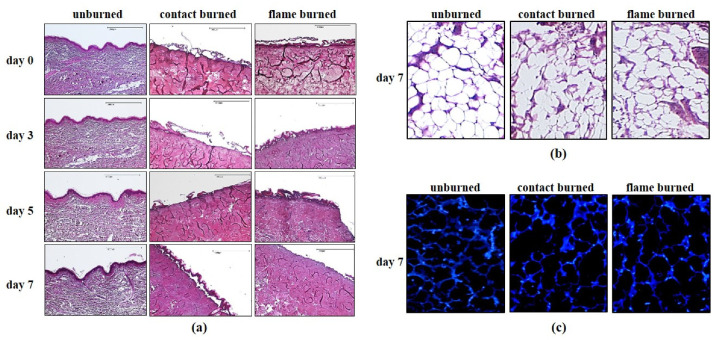
Comparison of tissue damage caused by contact and flame burns. Composite tissue samples from 6 different donors were subjected to contact (contact burned) burns or flame burns (flame burned) or were left untreated (unburned). Samples were either preserved immediately after injury (day 0) or further maintained in vitro for three (day 3), five (day 5), or seven days (day 7). (**a**) Tissue sections were analyzed using hematoxylin and eosin (HE) staining and viewed directly under a microscope. The images are from a single donor and are representative of 5 other healthy donors. The scale bar represents 1000 μm. (**b**) Magnified images (600 × 800 μm) of unburned or burned (contact or flame) HE-stained human subcutaneous adipose tissue sections 7 days after injury. (**c**) Tissue sections were analyzed using DAPI staining and observed under a fluorescence microscope. The images are from a single donor and representative of 5 other healthy donors.

**Figure A1:**
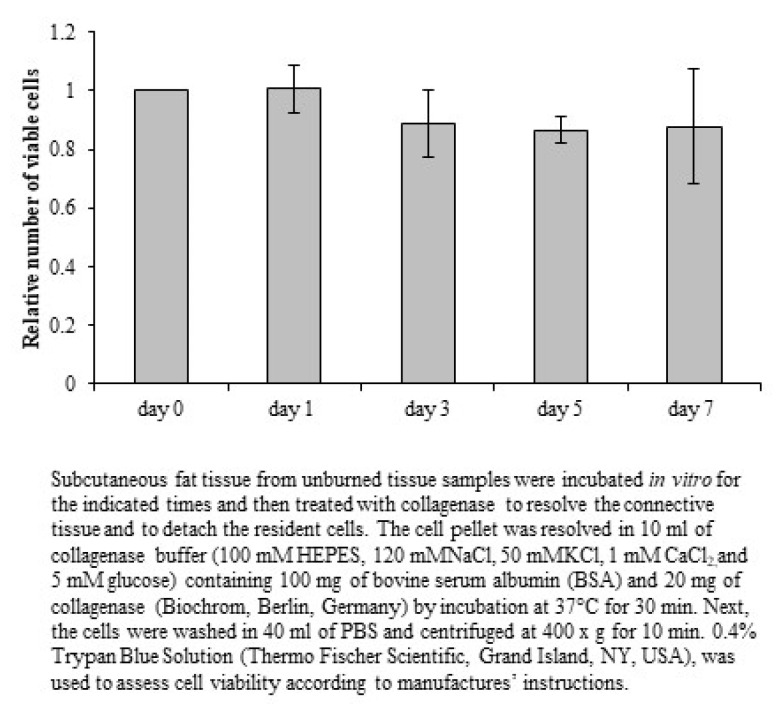
Viability of unburned tissue in vitro.

Previous clinical studies reported that tissue loss after burn injury can occasionally expand from the outer layers of human skin to deeper subcutaneous layers days after injury [14]. Although this phenomenon has not been fully elucidated, the circumstances of burn exposure are likely to play an important role. We previously established a human composite tissue model to investigate the response of subcutaneous adipose tissue to flame burns within 7 days following the burn injury [13]. Previous results confirmed the viability of tissue cells within 7 days in vitro [Figure A1 in Appendix A]. Here, we used this experimental model to compare the expansion of tissue damage caused by exposure to a flame applied by a flat-flame McKenna burner at 300 °C for 15 s or three times by repeated contacts to a steel bar at 100 °C for 30 s, which was previously tested to be the lowest limit for burn damage in our model (data not shown). There were six healthy donors between 27 and 49 years old included in this study (Supplementary Figure S1). For each donor, two parallel sets of experiments were performed for flame and contact burns. Each set of experiments consisted of four separate tissue sections, which were either preserved immediately after burn exposure (day 0) or maintained for 3, 5, or 7 days following exposure and then preserved (Figure 1a, day 3, 5 or 7). Multiple sections were evaluated using HE staining. The dimensions of the thermal damage of the superficial dermis became obvious in comparison to the unburned areas in each sample (Figure 1a). We observed strong deformation of collagen structures when focusing on the burn-damaged zone (10-mm-diameter) but not in the unburned zones (Figure 1a, contact and flame burned vs. unburned). The collagen structure was unchanged at 7 days after exposure to flame or contact burns, indicating that the thermal deformation of collagen was irreversible during this time (Figure 1a). The damage in the underlying subcutaneous adipose tissue became visible at higher magnifications (Figure 1b). Compared to the intact network of adipocytes in the unburned zone (Figure 1b, unburned), the burned zones exhibited destruction of the subcutaneous network of adipocytes (Figure 1b, contact or flame burned). Next, we used DAPI staining to differentiate apoptotic and nonapoptotic cells. We observed a large number of intact and nonapoptotic cells within the damaged network of subcutaneous adipocytes at seven days postburn injury (Figure 1c). These data indicated that the flame and contact burns inflicted similar structural damage in human cutaneous and subcutaneous adipose tissues.

**Figure 2:**
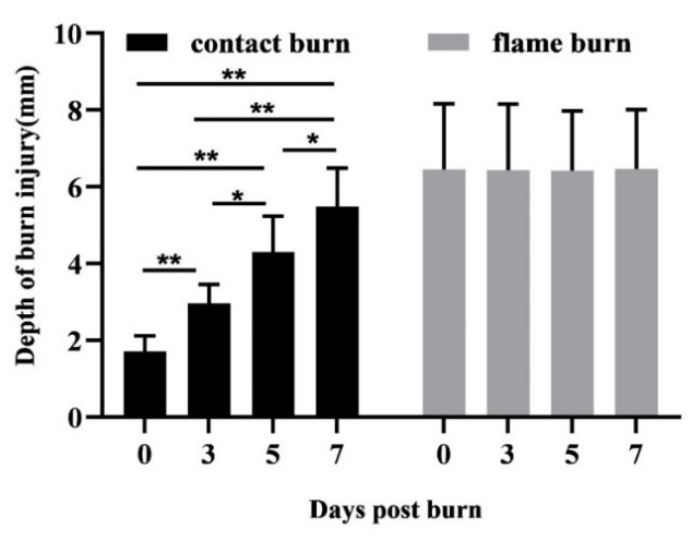
Depth of tissue damage after contact and flame burns. Composite tissue samples from 6 different donors were subjected to contact (contact burned) or flame burns (flame burned). Samples were preserved immediately after injury (day 0) or further maintained in vitro for three (day 3), five (day 5), or seven days (day 7). Later, tissue sections were analyzed using HE staining and viewed directly under a microscope. The chart shows the mean depth (0–10 mm) of the burn-damaged zones. Damaged tissue was identified by the destruction of the adipocyte network based on microscopic evaluation of the HE-stained samples. The burn damage depth was defined as the distance from the most distally detected tissue damage to the most superficial core of the burned epidermis. Statistical significance is shown with * *p* ≤ 0.05 or ** *p* ≤ 0.01.

To compare the expansion of tissue damage after contact and flame burns, we measured the maximum distance between the center of the burn site and the deepest visible adipose tissue damage in HE-stained tissue specimens. The data for contact burns (black bars) and flame burns (gray bars) were summarized in a single chart (Figure 2). Immediately after injury exposure (day 0), the mean depth of tissue damage reached 1.71 mm after contact burns and 6.45 mm after flame burns. During the seven-day period, the depth of tissue damage did not significantly change after flame burns. However, we observed a continuous expansion of tissue damage deeper in subcutaneous adipose tissue up to seven days after contact burns. Thus, the feature of thermal forces can directly influence the time-dependent progression of subcutaneous tissue damage.

### 3.2. Activation of Subcutaneous Monocytes and Fibroblasts by Adipose Tissue Damage

Macrophages represent the most abundant class of immune cells in subcutaneous adipose tissue. They are activated by tissue damage and play a crucial role in the clearance of dead cells. There were three specific markers, CD68, CD80 and CD163, established to identify resident monocytes, M1 and M2 macrophages, respectively. Tissue damage also activates subcutaneous fibroblasts and myofibroblasts that facilitate tissue regeneration and wound healing. HSP47, a 47-kDa heat shock protein, is a collagen-specific molecular chaperone and a specific marker for fibroblasts and myofibroblasts [15,16].

**Figure 3:**
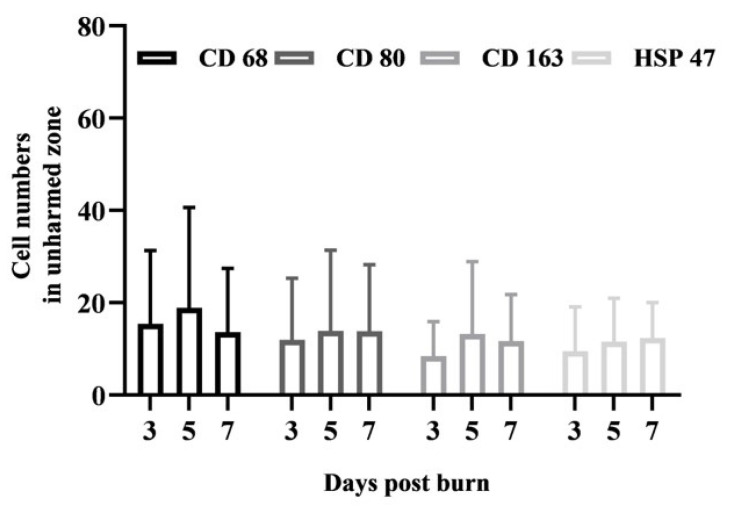
Number of resident macrophages and fibroblasts in undamaged subcutaneous tissue. Composite tissue samples from 6 different donors were maintained in vitro for three, five or seven days after injury. Later, tissue sections were subjected to immunohistochemistry and diaminobenzidine (DAB) staining using antibodies against CD68, CD80, CD163, and HSP47. The number of positive cells was determined in at least 5 equivalent microscopic fields in different locations within the undamaged zones of all the samples. The mean numbers of positive cells from days 3, 5, and 7 are shown, and the error bars indicate the standard deviation (SD).

**Figure 4:**
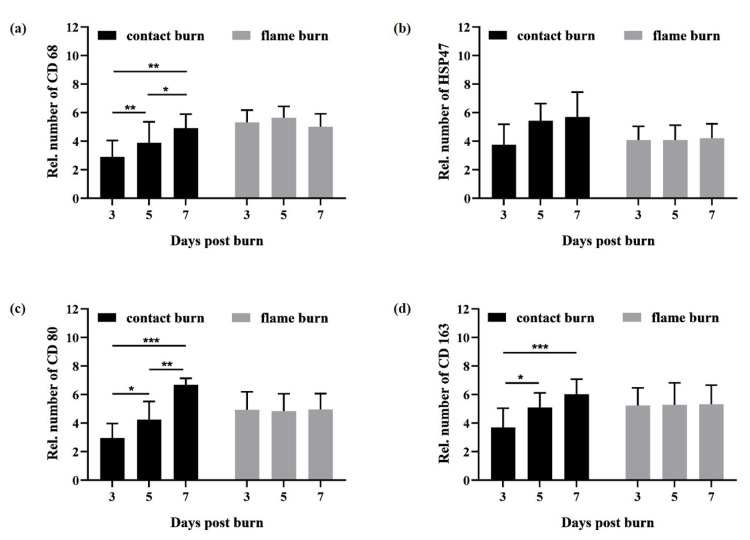
Number spatiotemporal increase in the number of macrophages and fibroblasts in response to contact and flame burns. Composite tissue samples from 6 different donors were subjected to contact (contact burn) or flame burns (flame burn). Samples were maintained in vitro for three (day 3), five (day 5), or seven days (day 7) after injury. Later, tissue sections were subjected to immunohistochemistry and DAB staining using antibodies against CD68, HSP47, CD80 or CD163. The number of positive cells was determined in at least 5 equivalent microscopic fields in different locations within the burn-damaged and undamaged zones of subcutaneous tissue. To calculate the relative number of cells, the mean number of positive cells in the unharmed zone was set to 1 and was compared to the number of positive cells in the burn-damaged zone. The diagrams summarize the mean relative numbers of positive cells expressing CD68 (**a**), HSP47 (**b**), CD80 (**c**) or CD163 (**d**). The error bars indicate the standard deviation (SD). Statistical significance is shown with * *p* ≤ 0.05, ** *p* ≤ 0.01 or *** *p* ≤ 0.001.

**Figure A2:**
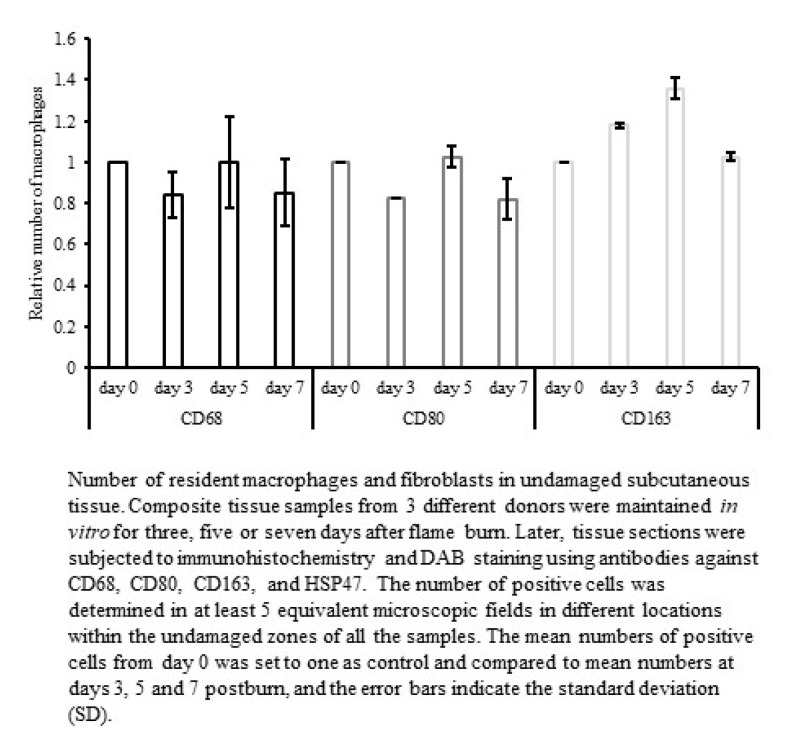
Number of macrophages in unharmed zones.

Using our human composite tissue model in a previous study, we observed no significant alterations in macrophages in burned zones until 3 days and in unharmed zones until 7 days postburn (Figure A2 in Appendix A) [13]. Here, we used antibodies against CD68, CD80, CD163, and HSP47 to examine whether the activation of resident macrophages and fibroblasts may correspond with the differential expansion of tissue damage after contact and flame burns. We analyzed a total of 576 sections, including three replicate sections from each donor, and recorded observations relating to antibody binding, burn condition at 3, 5, or 7 days postburn. First, we determined the number of macrophages and fibroblasts within five distinct but equivalent microscopic fields of unharmed zones in all sections (Figure 3). Despite variations among the donors, no significant differences were observed in unharmed zones of parallel sections from the same donor. Moreover, the mean number of macrophages and fibroblasts did not significantly change from day 3 to 7 during in vitro maintenance (Figure 3). These results emphasized the specificity of the local increase in macrophages and fibroblasts in burn-damaged zones. Because the number of cells may vary between separate sections from different donors, we determined the relative change in the number of cells in burn-harmed zones compared to the corresponding unharmed zones in each tissue slide. Both contact and flame burns led to a significant increase in macrophages and fibroblasts in the burn-damaged zone compared to the unharmed zone within three days (Figure 4a–d, Supplementary Tables S2–S4). Strikingly, the increase in macrophages and fibroblasts remained unaffected within seven days after flame burns (Figure 4a–d, gray bars). After contact burns, however, the numbers of macrophages and fibroblasts in burn-damaged zones were significantly increased compared to those in the unharmed zones from day 3 to day 7 (Figure 4a–d, black bars). In fact, the mean increases in the numbers of CD80-, CD163- or HSP47-positive cells by contact burns were higher than those by flame burns after 7 days (Figure 4b–d, day 7). Taken together, these results suggested that subcutaneous tissue damage and the subsequent activation of macrophages and fibroblasts followed a progressive course after contact burns but not after flame burns.

## 4. Discussion

Burn wound progression refers to an unsolved phenomenon in the clinic that can induce the conversion of a burn wound from a superficial burn to a deep subcutaneous burn with severe consequences for burn victims. In current clinical practice, burn wounds are initially evaluated only for extent, depth, and circumferential components [2]. All decisions regarding the wound care, hospitalization, and transfer are made based on this information. The main findings of our study suggest that thermal forces decisively influence burn wound progression in human subcutaneous tissue within days after injury. Thus, the type of burns and their thermal characteristics, like heath source and duration, may be relevant to initial decision-making in primary care. In our experimental model, adipose tissue damage and macrophage and fibroblast activation progress together with analog changes in spatiotemporal dynamics. Furthermore, macrophage and fibroblast activation is a hallmark of inflammation and tissue regeneration, suggesting that both processes are initiated within the damaged network of subcutaneous adipose tissue immediately after burn injury. This highlights the crucial role of subcutaneous adipose tissue in human burn wound progression.

A study of tissue samples from burn victims two to six days postburn suggested that a higher proinflammatory tissue status can explain the high conversion rate from partial to full thickness burns [17]. Notably, it is not feasible to capture details on thermal burn conditions upon clinical admission, and tissue assessments generally occur at the wound site days after injury. Previous studies revealed that circulating macrophages infiltrate into the wound between 24 h and 46 days after burns and thereby obscure the true inflammatory state of the damaged tissue [18]. In our model, we were able to assess the tissue before and immediately after the onset of damage in samples from six donors. Among them, two donors exhibited between 2- and 11-fold higher numbers of subcutaneous macrophages, indicating a higher proinflammatory tissue status before burn exposure (data not shown). However, the progression of tissue damage in both donors was not significantly different than that in other donors. Although our donor collection did not encompass a multitude of different inflammatory preconditions, our experimental model implies that the dynamics of wound progression in contact burns are independent of the inflammatory tissue environment. Moreover, contact and flame burns inflicted distinct dynamics of wound progression in the same donor. Therefore, we assume that the inflammatory environment at the injury site has a minor impact on burn wound progression.

Previous studies have suggested that oxidative stress and apoptosis play important roles in prolonged inflammation and tissue damage [19]. However, as many studies have used animal burn models, there is a strong limitation to the clinical applicability of the results on human burns [1]. Among many reported animal burn models, a porcine model has reported burn wound progression up to 7 days postburn [20]. Scald burns conferred by temperatures from 50 °C to 90 °C inflicted visible damage on the dermis, which progressed to a depth of 3 mm 7 days postburn. However, no harm to the underlying adipose tissue was detected in the porcine model. We observed that contact burns at 100 °C inflicted significant progressive damage on human subcutaneous adipose tissue. This discrepancy is either due to the difference between 90 °C and 100 °C or to interspecies differences in the epidermal thickness and the vascular network between porcine and human models, despite their well-recognized translational relevance. Thus, the human composite tissue model is a useful approach used to investigate the local impact of thermal forces on the human dermis.

A large number of macrophage markers have been identified in murine models, but the majority of them have not been translated to humans [8,21]. The current classification of human macrophages is based on few protein markers from blood-derived monocytes, which differentiate into M1- and M2-type macrophages in vitro [8,21]; however, further studies revealed that the M1/M2 paradigm may be misleading during acute tissue injury, when circulating macrophages infiltrate the tissue and exceed the number of resident macrophages [22,23]. Thus, our human composite tissue model is undoubtedly subjected to the same limitations as the M1/M2 paradigm [24,25], but the migration of circulating blood-derived macrophages could be excluded, thus allowing the number of macrophages to remain constant in the unharmed zone of all samples (Figure 3). Therefore, the use of M1/M2 polarization may be limited to the well-defined in vitro conditions as in the human tissue burn model. Independent of the possibly disparate inflammatory functions of M1 (CD80+) and M2 (CD163+) macrophages, both subtypes emerge simultaneously at the site of adipose tissue damage. Furthermore, the total number of all macrophages (CD68+) increases at the site of injury. We assume that preconditioned M1 and M2 subtypes reside within the adipose tissue and become independently activated in response to damage to mature adipocytes. Macrophages maintain adipose tissue by eliminating dead adipocytes, but they can also regulate the inflammatory microenvironment during weight loss or obesity and excessive accumulation of fat [4,26,27,28].

Human fibroblasts represent a complex group of cells with distinct functions in the regulation of tissue matrix deposition and likely serve as pluripotent mesenchymal stem cells [29]. Previous reports suggested that tissue regeneration follows the sequential activation of pro- and anti-inflammatory signals that ultimately activate fibroblasts and their proliferation [9,30]. Our results show that the activation of fibroblasts and polarization of M1 and M2 macrophages occur simultaneously in the same spatiotemporal dynamics. Thus, activation of subcutaneous fibroblasts may participate or be a part of the inflammatory response at the early stages of wound healing.

## 5. Conclusions

We conclude that the depth of tissue damage largely depends on the type of thermal insult in our human burn model. The injuries can progress different dynamics in the inflammatory response and tissue repair at the time of injury. Tissue-resident macrophages play diverse roles in the development and targeting of acute responses to injuries and tissue repair. Thus, a more precise typing of tissue-resident macrophage subpopulations is crucial for future therapies. The human composite tissue model is a resourceful approach for the characterization of human tissue macrophage populations. This model, when utilized for contact burn experiments, provides dynamic information relating to injury and tissue repair, which allows the study of the source and nature of signals that mediate macrophage polarization.

## Data Availability

All data except patients’ information will be made available upon request.

## Supplementary Materials

The following are available online at https://www.mdpi.com/2079-7737/10/1/40/s1, Figure S1: Experimental setup, Table S1: The relative cell numbers of CD68 in contact and flame burns, Table S2: The relative cell numbers of CD80 in contact and flame burns, Table S3: The relative cell numbers of CD163 in contact and flame burns, Table S4: The relative cell numbers of CDHSP47 in contact and flame burns.
