# Supplementary figures and images for: Experimental Study of Burn Damage Progression in a Human Composite Tissue Model
Source: Biology (Basel). 2021 Jan 8;10(1):40. doi: 10.3390/biology10010040 (PMC7827328; doi:10.3390/biology10010040)

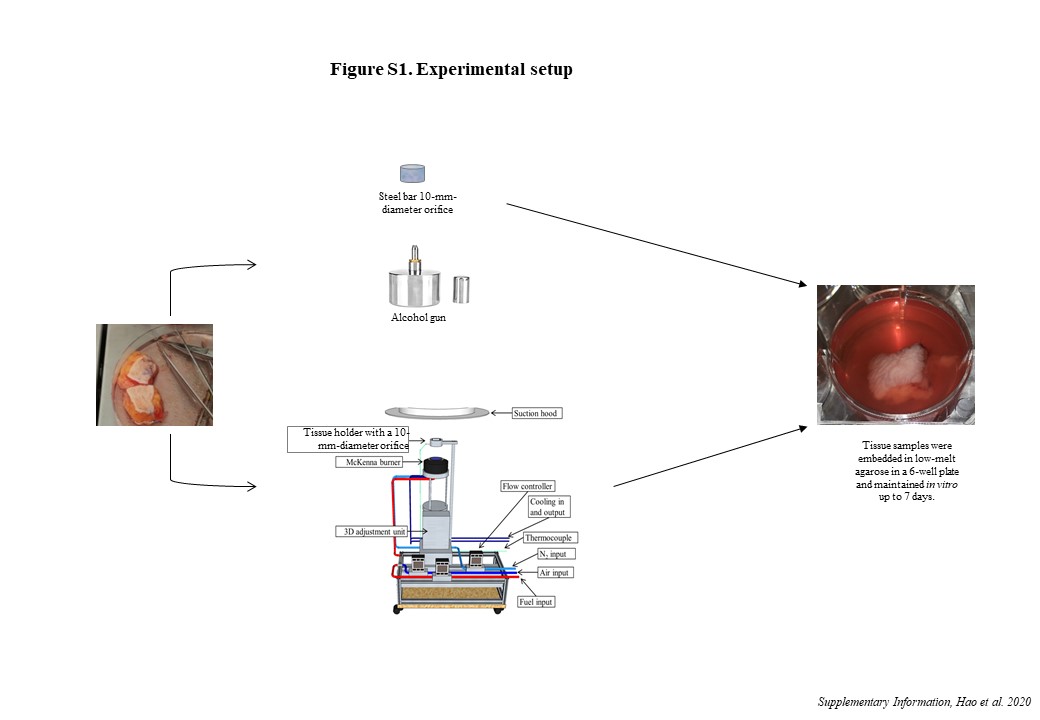

Supplement: Supplementary file 1 [file biology-10-00040-s001.zip › Supplementary FigS1.JPG]

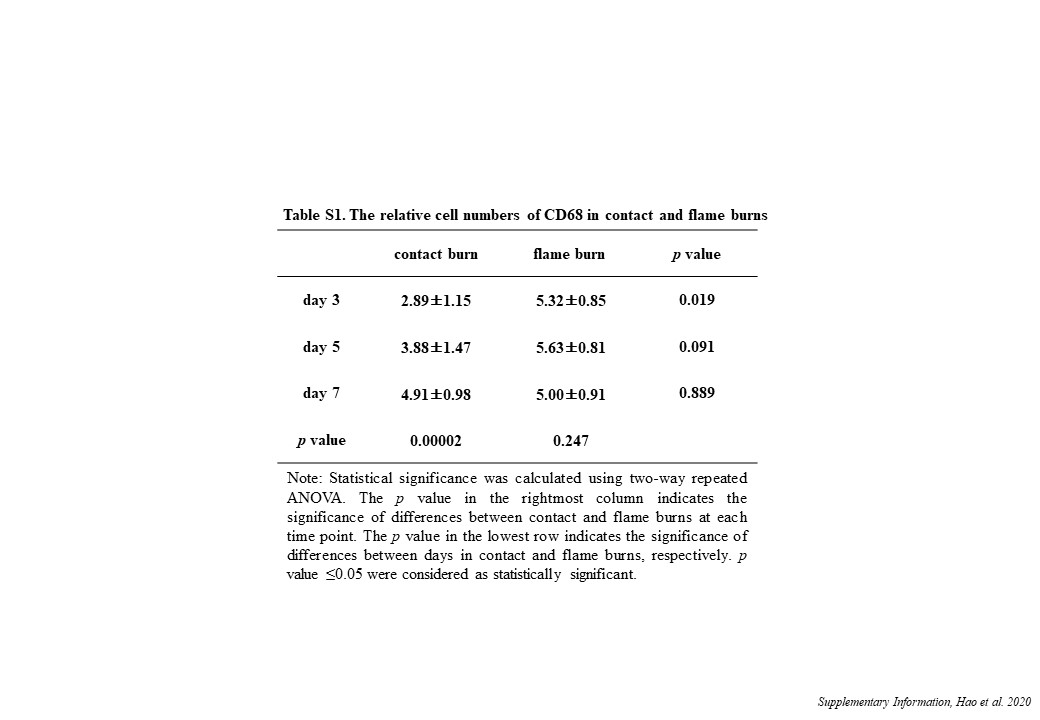

Supplement: Supplementary file 1 [file biology-10-00040-s001.zip › Supplementary TabS1.JPG]

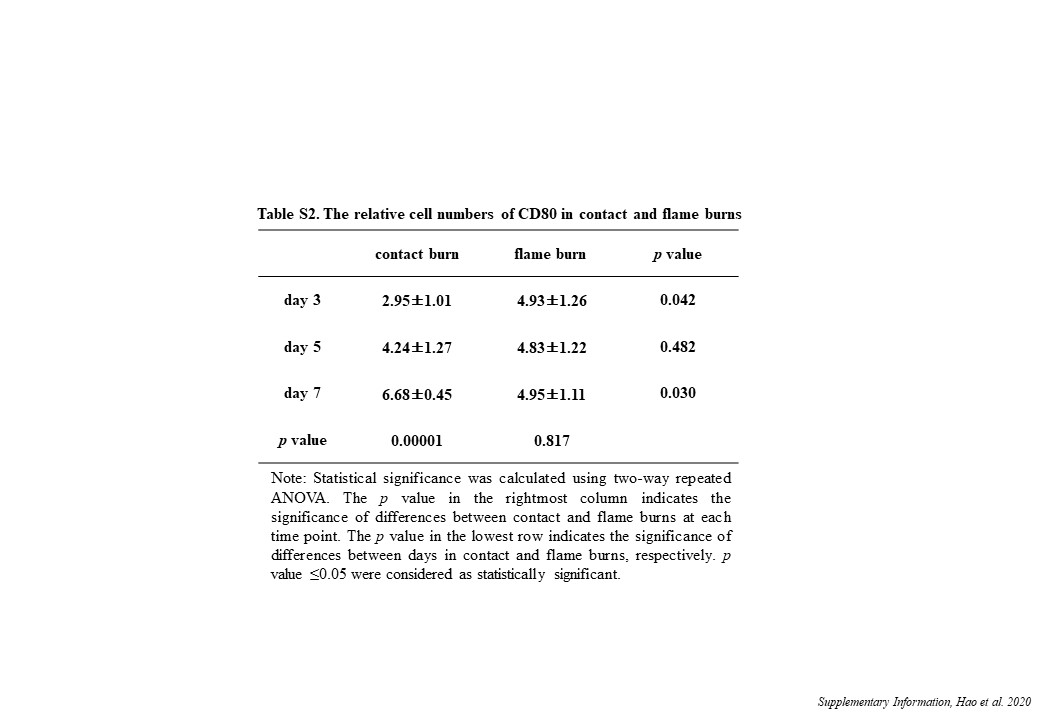

Supplement: Supplementary file 1 [file biology-10-00040-s001.zip › Supplementary TabS2.JPG]

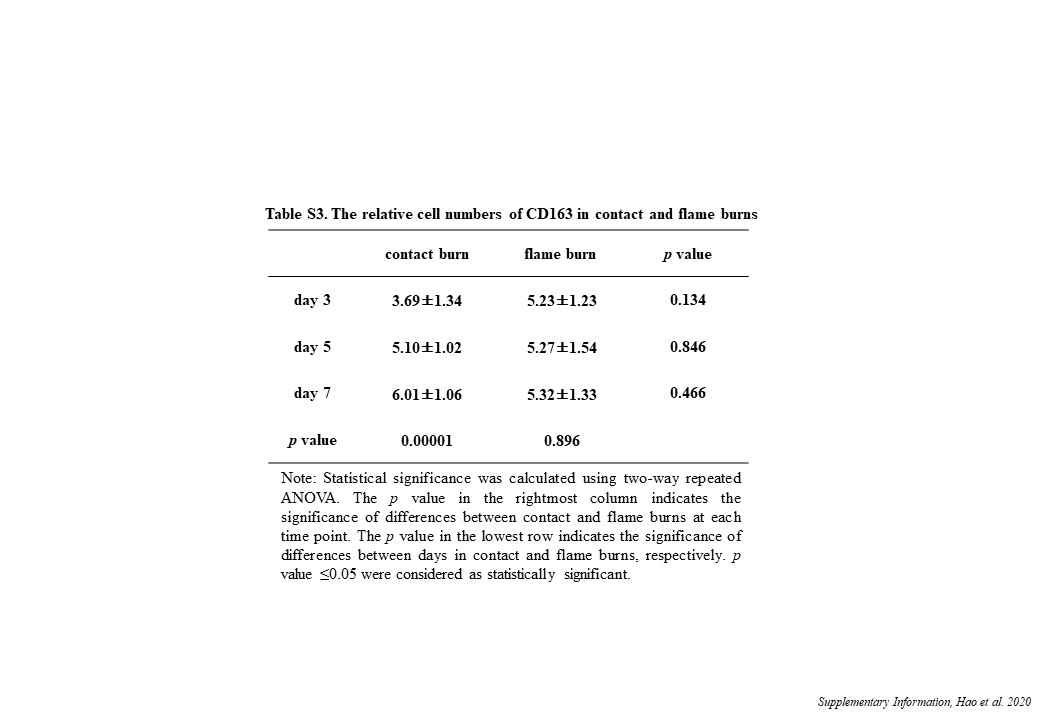

Supplement: Supplementary file 1 [file biology-10-00040-s001.zip › Supplementary TabS3.JPG]

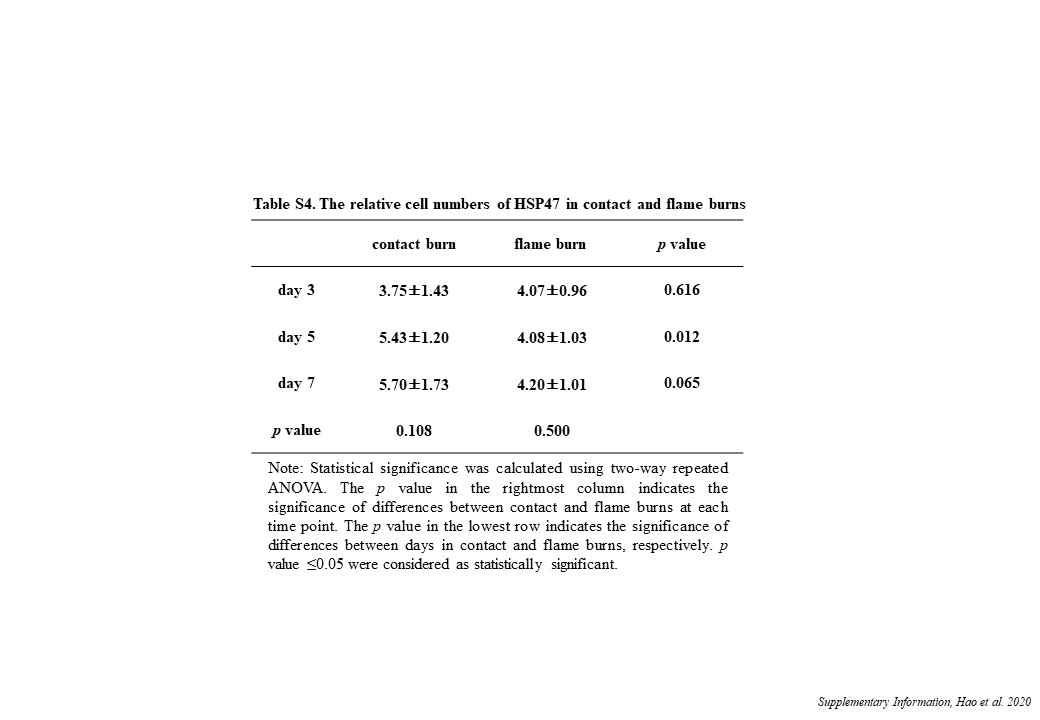

Supplement: Supplementary file 1 [file biology-10-00040-s001.zip › Supplementary TabS4.JPG]
